# A one health visual analytics approach for syndromic surveillance^☆^

**DOI:** 10.1016/j.onehlt.2026.101561

**Published:** 2026-10-14

**Authors:** Aseel Basheer, Said Jalal Saidi, Parisa Masnadi Khiabani, Wolfgang Jentner, Aaron Wendelboe, Jason Vogel, Katrin Kuhn, Michael C. Wimberly, Jo Ann Morrison, Kseniya Topdjian, Dean Hougen, David Ebert

**Affiliations:** a University of Oklahoma, Norman, 73019, OK, USA; b University of Arkansas for Medical Sciences, Little Rock, 72205, AR, USA; c University of Oklahoma Health Campus, Oklahoma City, 73104, OK, USA; d University of Arizona, Tucson, 85721, AZ, USA; e Mars Veterinary Health, Vancouver, 98684, WA, USA; f Banfield Pet Hospital, Vancouver, 98684, WA, USA

**Keywords:** One Health, COVID-19, Epidemiology, Visual analytics, Time series prediction, Anomaly detection

## Abstract

Effective syndromic surveillance requires integrating diverse data streams while maintaining transparency and interpretability to support timely and informed public health action. Traditional surveillance approaches often rely on single-domain data, such as clinical reports or environmental measurements, or employ black-box models that limit stakeholder trust and validation. To address these challenges, we present a One Health visual analytics framework that integrates heterogeneous surveillance signals with complementary predictive models and interactive model analysis, enabling systematic comparison of data contributions, model configurations, and predictive uncertainty across infectious disease surveillance tasks. The framework integrates mixed-effects models, Transformer-based deep learning architectures with spatiotemporal embeddings, and interactive dashboards to balance predictive performance with explainability in a white-box environment. We demonstrate the framework’s utility through three mixed-effects case studies and the complementary Transformer forecasting experiment: (i) prediction of influenza and RSV cases in Arizona, (ii) modeling of COVID-19 cases and deaths in Arkansas, and (iii) prediction of COVID-19 hospitalizations in Oklahoma using wastewater, animal health, and environmental data. Across the case studies, integrating multi-source signals improved predictive accuracy for most configurations, although the incremental value of individual streams proved species- and configuration-dependent, while hierarchical random effects (e.g., county, month, age group) captured contextual variation and enhanced robustness. For longer-term forecasting, Transformer models with spatial and spatiotemporal embeddings outperformed baseline statistical models, highlighting the value of contextual information. By transforming complex modeling outputs into interpretable visual tools, the framework supports proactive decision-making, enhances public health preparedness, and strengthens One Health collaboration across human, environmental, and animal health domains. This work introduces a generalizable One Health approach for transparent, data-driven syndromic surveillance with direct implications for health security and community resilience. The novelty of this work is the integration of heterogeneous One Health signals with complementary modeling strategies and interactive model provenance so that prediction and interpretation can be examined together across health domains.

## Highlights

• One Health framework unifies clinical, wastewater, and environmental data.
• Mixed-effects and Transformer models improve outbreak forecasting.
• Multi-source datasets boost predictive accuracy across case studies.
• Spatiotemporal Transformers outperform ARIMA for long-term forecasts.
• An interactive dashboard enables transparent and explainable surveillance.

## Graphical abstract

**Figure:**
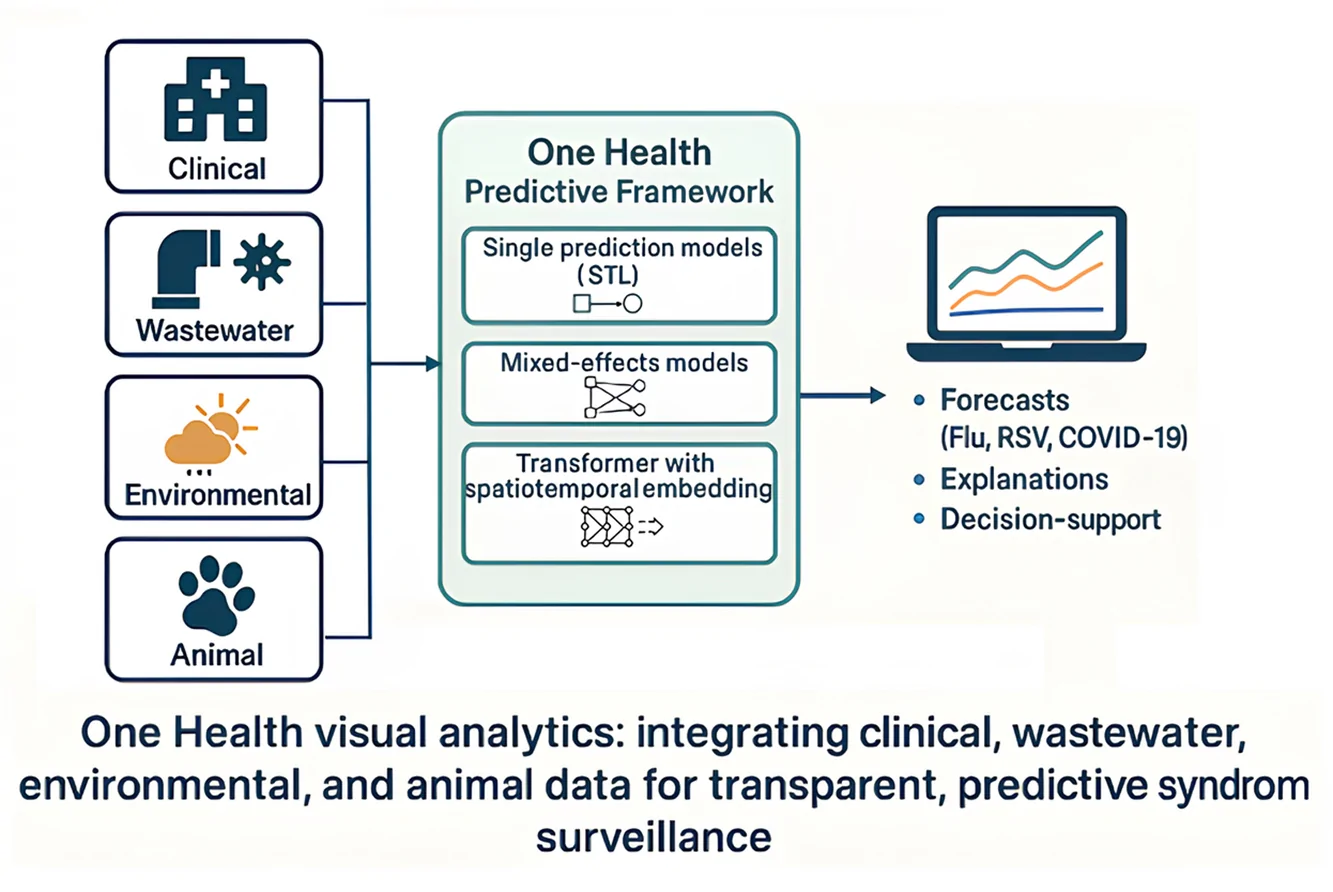

## 1 Introduction

Recent public health crises, including the COVID-19 pandemic, have underscored the pressing need for predictive systems that can integrate diverse data sources and provide actionable insights for infectious disease management [1], [2]. Infectious disease transmission is inherently multifactorial, influenced by human behavior, social interactions, population density, environmental conditions such as climate and air pollution, and interactions with animal reservoirs [3], [4]. Understanding these complex dynamics is crucial for effective syndromic surveillance and pandemic preparedness, particularly within the *One Health* paradigm, which emphasizes the interconnections between human, animal, and environmental health [5], [6].

Despite the availability of large-scale datasets, most existing models remain limited; they often rely on single-source data (e.g., clinical counts or wastewater surveillance) or black-box methods that hinder interpretability [7], [8]. A systematic review of 202 One Health systems published between 2015 and 2024 found that 20% draw on human data alone, 12% on animal data alone, and 10% on environmental data alone, while only 6% support prediction or forecasting and just 16% employ machine learning or artificial intelligence at all [9]. Similarly, current surveillance systems rarely provide interactive tools for exploring multivariate relationships or validating predictions in real-time [10], [11]. This creates a critical gap: the need for integrated, explainable, and interactive platforms that enable experts to explore cross-domain data and assess predictive models comprehensively.

Syndromic surveillance can be enhanced by integrating theoretical insights into disease transmission with advanced modeling and interactive visual analytics. Environmental factors (temperature, precipitation, air quality), social determinants (population density, mobility), and animal reservoirs all contribute to disease spread [1], [3]. In addition to these environmental and societal drivers, urban density, mobility networks, and socioeconomic disparities amplify transmission dynamics, shaping disease risk and outcomes across populations. Such heterogeneity in exposure and vulnerability underscores the importance of combining epidemiological, environmental, and behavioral data for accurate modeling and timely intervention [12], [13].

*Visual analytics*, which is “the science of analytical reasoning facilitated by interactive visual interfaces”, enables exploration of such complex, multivariate data and supports human decision-making alongside automated analysis [14], [15]. Linear mixed-effects models provide interpretable relationships, while deep learning architectures (e.g., Transformers) capture complex nonlinear dependencies and serve as performance benchmarks [16].

Although visual analytics and human-designed models, such as mixed-effects models significantly contribute to understanding relationships, computationally intensive approaches, like deep learning, can model complex nonlinear relationships and serve as a benchmark for predicting the available data. Building on the complementary strengths of these modeling approaches, this paper presents a One Health visual analytics framework that integrates clinical health records, wastewater monitoring, and environmental indicators into a unified predictive intelligence platform. Our framework combines mixed-effects models, deep learning architectures (e.g., Transformer [16]), and interactive dashboards, supporting both predictive accuracy and interpretability. Central to the design is its white-box orientation: the framework uses interactive visualizations, tooltips, hover-based data inspection, and exploratory filtering to allow users to directly examine model inputs, patterns, and outputs. These interactive capabilities are implemented using Dash and Plotly, providing an accessible interface for exploring model behavior and supporting explainability as a crucial requirement for responsible infectious disease modeling under the One Health paradigm. The study addresses the following research questions:

1. How can diverse One Health data streams be integrated to improve real-time prediction and situational awareness of infectious disease outbreaks?
2. What hybrid modeling strategies (single-variable regression, mixed-effects models, and deep learning) best balance interpretability and predictive accuracy?
3. How can interactive visual analytics enhance explainability, validation, and decision-making in pandemic preparedness?

The central novelty of this work is a unified One Health visual analytics framework that connects heterogeneous surveillance data, complementary predictive models, and interactive model analysis within a single workflow. Rather than treating human, animal, environmental, and wastewater surveillance as independent data sources or focusing on a single forecasting method, the framework enables these signals to be integrated, compared, and evaluated according to their contribution to infectious disease prediction. The framework further links model development with interactive visual analytics, allowing users to examine predictor outcome relationships, temporal and spatial heterogeneity, model alternatives, and predictive uncertainty while preserving the provenance of modeling decisions.

The contributions of this work are threefold:

• *An integrated One Health modeling framework:* that harmonizes human clinical, wastewater, environmental, and animal health surveillance signals and supports multiple complementary modeling strategies, including classical/decomposition-based forecasting, nonlinear mixed-effects models, and Transformer-based spatiotemporal forecasting.
• *A comparative modeling and evaluation workflow:* that enables systematic examination of how temporal, spatial, environmental, wastewater, and animal-health signals contribute to predictive performance across different infectious-disease outcomes and forecast horizons.
• *An interactive visual analytics layer:* that exposes predictor-outcome relationships, model configurations, performance metrics, uncertainty, and modeling provenance, allowing analysts to compare alternative models and interpret their results within a transparent One Health surveillance workflow.

## 2 Related work

Syndromic surveillance provides an important foundation for early infectious disease detection by monitoring symptoms, clinical encounters, and other probable health indicators before laboratory-confirmed diagnoses become available [17]. Rather than relying exclusively on confirmed diagnoses, syndromic surveillance tracks groups of symptoms and healthcare utilization patterns that may indicate emerging health threats [18]. For example, increases in influenza-like illness (ILI) or emergency department visits can provide early indications of potential outbreaks before laboratory confirmation [19]. This enables public health agencies to initiate investigation and control measures more rapidly [17]. However, syndromic surveillance alone provides limited insight into the broader environmental, spatial, and ecological conditions that may influence disease dynamics [18]. Infectious disease modeling complements surveillance by providing mathematical and computational approaches for characterizing disease transmission and projecting future outcomes. Compartmental models such as SEIR categorize populations according to disease states and provide a foundation for representing transmission dynamics [20]. More approaches incorporate temporal, spatial, and spatiotemporal information (e.g. autoregressive models, recurrent neural networks, geographic information systems, and other predictive techniques [18]). These models became particularly important during the COVID-19 pandemic for informing intervention timing, resource allocation, and vaccination strategies [1], [4].

The limitations of single-domain surveillance have motivated increasing interest in *One Health* approaches, which recognize that human, animal, and environmental health are interconnected and that infectious disease emergence can result from interactions across these domains [6]. One Health surveillance therefore seeks to integrate information from multiple health domains to provide a more comprehensive representation of disease risk and transmission. Such systems can combine human and animal health data with environmental observations to improve disease detection, characterize zoonotic risks, and support coordinated intervention strategies [21]. Environmental surveillance is particularly important within this framework because it encompasses both direct pathogen detection in environmental media and monitoring of environmental conditions that may influence disease dynamics. These data can include pathogen measurements from wastewater, soil, air, and surfaces, as well as environmental risk factors such as air pollution and particulate matter [3]. Wastewater-based epidemiology, for example, can provide an early signal of community transmission, while environmental conditions may provide additional context for interpreting changes in disease incidence [3]. Despite this potential, integrating heterogeneous data streams across One Health domains remains challenging because the data differ in spatial and temporal resolution, measurement characteristics, and epidemiological meaning. A recent systematic review of One Health systems for emerging infectious disease detection and management found that monitoring remains the dominant system purpose (56%), whereas prediction and forecasting account for only 6% of identified systems [9]. The review also found that human-animal integration is the most common multi-domain combination, while systems simultaneously integrating human, animal, and environmental data remain comparatively scarce [9]. These findings highlight a gap between the conceptual emphasis on integrated One Health surveillance and the limited availability of operational systems that combine heterogeneous data for predictive decision support.

Machine learning (ML) provides complementary capabilities for extracting patterns and generating predictions from complex epidemiological data. Traditional ML algorithms such as Random Forest [22] and Support Vector Machines [23] have been applied to classification and prediction tasks, while Isolation Forest supports anomaly detection for identifying unusual patterns in surveillance data [24]. Recurrent neural networks such as Long Short-Term Memory (LSTM) models can learn temporal dependencies in sequential epidemiological observations [25]. More broadly, ML and predictive modeling approaches have increasingly been applied to heterogeneous temporal and spatiotemporal health data [7]. For example, mixed-effects models have been used to predict COVID-19 hospitalizations from wastewater surveillance measurements, integrating environmental indicators with epidemiological outcomes [26]. Forecasting approaches can also combine predictions from multiple epidemic models to characterize alternative disease trajectories and support outbreak management under uncertainty [27]. Epidemiological forecasting consequently extends surveillance from retrospective monitoring toward prospective assessment of disease dynamics. However, forecasting multiple outcomes from heterogeneous data sources introduces additional challenges related to model uncertainty, temporal dependencies, spatial variation, and differences among predictive models. Ensemble approaches can address some of these challenges by combining outputs from multiple models to provide more robust forecasts and characterize alternative predictions [27], [28].

Visual analytics provides an important interface between these computational methods and epidemiological decision-making by enabling users to interactively explore complex spatial, temporal, and predictive information. Epidemiological visual analytics systems commonly use interactive maps, charts, and dashboards to reveal spatial and temporal patterns that may be difficult to identify from tabular data alone [29]. A recent scoping review identified web-based platforms, choropleth maps, and interactive charts as common approaches for representing spatial–temporal epidemiological data and communicating disease trends and predictions [29]. Visual analytics has also been used to support pandemic decision-making, including the exploration of epidemic forecasts, comparison of alternative models, and visual sensitivity analysis [30], [31], [32]. For example, EPIsembleVis enables users to visually analyze and compare prediction ensembles from multiple COVID-19 models [32], while other systems incorporate network visualization to identify potential infection clusters [2]. These approaches demonstrate the value of interactive visual representations for interpreting complex epidemiological predictions. However, existing systems often focus on individual data domains, specific analytical tasks, or particular stages of the surveillance and forecasting workflow. Consequently, there remains a need for integrated visual analytics frameworks that bring together heterogeneous One Health data, predictive modeling, uncertainty, and interactive exploration within a unified decision-support environment. The present work addresses this gap by integrating human, animal, and environmental data with predictive analytics and interactive visual exploration to support proactive infectious disease surveillance and decision-making.

## 3 Materials and methods

### 3.1 Proposed general approach

Our modeling approach supported flexible, transparent, and multi-scale prediction of infectious disease dynamics within a One Health framework. It integrated three complementary methodologies: (i) individual prediction models, which leverage classical and decomposition-based time-series methods to capture temporal trends and seasonality; (ii) linear mixed-effects models, which incorporate hierarchical and contextual random effects to account for county-level, demographic, and temporal heterogeneity while integrating multi-source predictors such as clinical data, wastewater surveillance, and environmental conditions [33]; and (iii) Transformer-based architectures [16] with spatiotemporal embeddings, which exploited both spatial and temporal correlations to improve medium- and long-term forecasting. Together, these approaches provided a hybrid framework that balances predictive accuracy, interpretability, and flexibility, enabling domain experts to explore, validate, and compare model outputs in a transparent One Health context. The general workflow is summarized in Fig. 1. It consists of four major stages:

• **Data Integration and Preprocessing:** Clinical outcomes, wastewater surveillance, and environmental indicators are harmonized at a weekly, county-level resolution. Missing data are imputed, variables are standardized, and temporal alignment is ensured across all sources.
• **Feature Engineering:** Derived meteorological metrics, including Net Effective Temperature (NET), along with lagged meteorological and epidemiological variables and polynomial terms, are computed to capture nonlinear and delayed effects.
• **Modeling and Forecasting:** Three complementary modeling strategies, single prediction models, mixed-effects models, and Transformer-based architectures, are employed to capture temporal patterns, multilevel heterogeneity, and nonlinear dependencies.
• **Interactive Visualization and Evaluation:** Model outputs and cross-domain relationships are explored through the Predictive Intelligence for Pandemic Preparedness (PIPP) dashboard and the Mixed-Effects Modeling Tool, enabling explainable and interactive analysis.

**Fig. 1:**
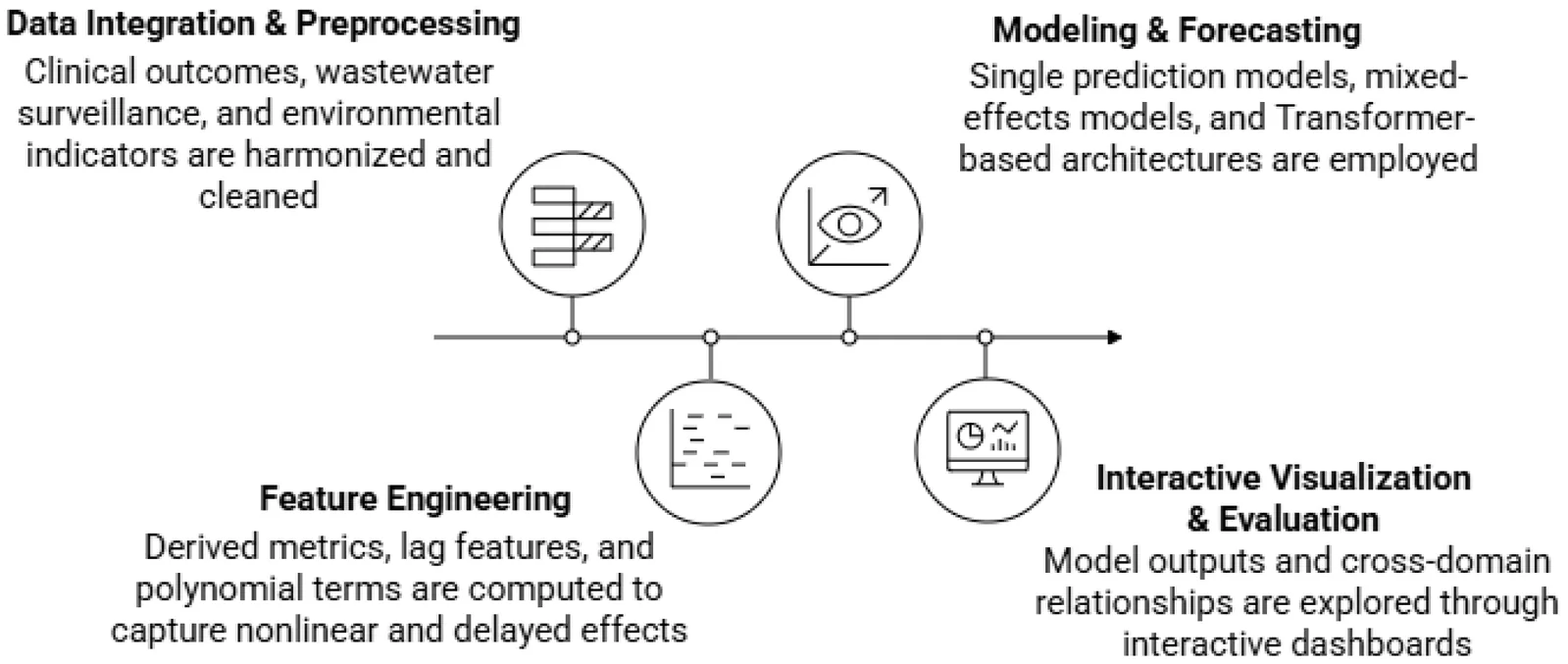
The four major stages of the proposed framework.

### 3.2 Data-stream contributions to prediction and interpretation

A central feature of the proposed One Health visual analytics framework is the integration of heterogeneous surveillance streams according to their distinct contributions to prediction and interpretation, rather than treating all variables as interchangeable predictors, as shown in Table 1. Across the case studies, human clinical outcomes, environmental and internet-based signals, wastewater surveillance, animal-health indicators, and spatial and temporal features provide complementary evidence about disease dynamics. The contribution of each stream depends on the surveillance stage, outcome, temporal context, and model configuration.

**Human clinical outcomes** provide the primary observed epidemiological signal against which predictive models are evaluated. Flu, RSV, COVID-19 cases, deaths, and hospitalizations represent realized disease burden and therefore serve as the principal target variables for assessing whether additional surveillance streams provide predictive value. These outcomes also provide the operational interpretation of model predictions, linking statistical associations to clinically meaningful changes in disease activity.

**NET and environmental data** provide contextual information about environmental and population-level conditions that may precede or coincide with changes in disease activity. In the Arizona and Arkansas case studies, NET was used as an environmental predictor, while temporal random effects captured recurring seasonal variation. The observed improvement associated with month effects indicates that environmental signals are not independent of temporal context; their predictive contribution depends on when and under what seasonal conditions they are observed.

**Wastewater surveillance** provides a complementary population-level signal that can capture pathogen activity independently of clinical testing and healthcare utilization. In the Oklahoma case study, wastewater measurements contributed additional predictive information when combined with NET and animal-health indicators. Its value therefore lies not only in predicting human hospitalization levels, but also in providing an independent surveillance stream that can complement clinical and environmental observations.

**Animal-health data** extend the framework beyond human-only surveillance by incorporating disease-related signals from companion animals. In Oklahoma, canine and feline syndromic indicators were evaluated alongside human hospitalization, wastewater, and NET data. The animal-health component in this study was therefore limited to companion animal species and did not encompass livestock or agricultural animal surveillance, which represents a distinct One Health context with different surveillance objectives and data characteristics. The contribution of companion animal data was configuration- and species-dependent, indicating that their inclusion should not be assumed to improve prediction universally. Instead, the framework enables their incremental predictive value to be evaluated in combination with other surveillance domains.

**Spatial features** capture geographic heterogeneity in disease transmission and surveillance conditions. In the Transformer case study, spatial embeddings improved forecasting performance, particularly when combined with temporal information for shorter forecast horizons and when used independently for the longer 21-day horizon. Spatial information therefore contributes both predictive structure and interpretability by identifying geographic dependencies that cannot be represented through temporal patterns alone.

**Temporal features** capture seasonality, persistence, lagged relationships, and changes in disease dynamics over time. Their contribution was evident across the mixed-effects case studies through month-level random effects and in the Transformer models through temporal embeddings. The results demonstrate that the predictive value of other streams is conditional on temporal context: the same environmental, wastewater, or spatial signal may have different implications depending on the time period and forecast horizon.

Together, these data streams support a multi-perspective representation of disease dynamics. The framework’s contribution is therefore not simply the aggregation of heterogeneous data, but the systematic assessment of how different surveillance domains contribute to prediction, contextual interpretation, and One Health decision-making. To capture the multifactorial nature of infectious disease dynamics, we employed diverse measures across the One Health domains: human, environmental, animal, and wastewater surveillance. Table 1 summarizes the primary role of each surveillance stream and its respective contribution to prediction and interpretation within the framework.

• **Clinical and Epidemiological Measures** For Oklahoma, weekly aggregated COVID-19 hospitalizations were obtained from the Oklahoma State Department of Health (OSDH) [34]. For comparative purposes, we also incorporated case and mortality data from the CDC [35] and influenza and RSV case counts from the Arizona Department of Health Services (ADHS) [36]. Clinical measures include: *COVID-19 hospitalizations, cases, and deaths* and *Influenza and RSV cases* (weekly counts). These variables represent human health outcomes and serve as dependent variables in predictive modeling.
• **Animal Health Surveillance Measures** To incorporate a complementary syndromic surveillance signal from the animal health domain, we use aggregated canine and feline veterinary records collected by Banfield Pet Hospital. The data include respiratory-related conditions such as coughing, kennel cough, upper respiratory disease, and pneumonia, summarized as weekly county-level veterinary activity. These data are incorporated as an additional population-level surveillance signal that may capture temporal patterns shared across companion animal and human respiratory health, environmental conditions, or community-level healthcare activity. Companion animals share household and community environments with humans and are therefore recognized as a relevant sentinel population within the One Health framework [37]. Their inclusion therefore allows the framework to evaluate whether animal health signals provide incremental predictive information when combined with human, wastewater, and environmental surveillance data.
• **Wastewater Surveillance Measures** Wastewater data quantify community-level concentrations of pathogens such as SARS-CoV-2, influenza, and RSV across multiple Oklahoma counties [38]. Key measures include: *Ct mean values* and *copies per mL* for targeted pathogens. Wastewater data act as early indicators of population-level infection trends, often preceding clinical reporting.
• **Environmental and Climatic Measures** Environmental parameters were derived from the *gridMET* dataset [39], which provides high-resolution daily meteorological data aggregated to the county level. Key variables include: *Daily mean, minimum, and maximum temperature (*T*) (°C)*, *Relative humidity (RH) (%)*, and *Wind speed (*v*) (m/s)*.
  Thermal comfort was summarized through the **Net Effective Temperature (NET)** index [40], calculated as:
  (1) $$\text{NET}=37-\frac{37-T}{0.68-0.0014\cdot RH+\frac{1}{1.76+1.4\cdot v^{0.75}}}-0.29\cdot T\cdot \left(1-0.01\cdot RH\right)$$
  NET integrates temperature, relative humidity, and wind speed into a measure of perceived outdoor thermal conditions. In this study, NET was included as a proxy for outdoor thermal comfort, with the rationale that extreme thermal conditions may influence human behavior and time spent indoors, potentially affecting patterns of respiratory disease transmission [12], [13].

**Table 1:** Data-stream contributions to prediction and interpretation across the One Health framework.

Data stream	Primary role	Contribution to prediction and interpretation
**Human clinical**	Observed disease burden	Provides the primary epidemiological outcomes used as prediction targets and anchors model results to clinically meaningful disease activity.

**NET/environmental**	Environmental context	Captures environmental conditions associated with disease variation; its predictive contribution depends on seasonal and temporal context.

**Wastewater**	Population-level pathogen signal	Provides an independent community-level signal that complements clinical surveillance and can improve prediction when combined with other streams.

**Animal health**	Cross-species surveillance	Extends surveillance beyond human populations and enables evaluation of whether canine/feline signals provide incremental predictive information.

**Spatial**	Geographic heterogeneity	Captures location-dependent disease dynamics and spatial dependencies that may not be represented by temporal information alone.

**Temporal**	Seasonality and disease dynamics	Captures seasonality, persistence, lagged relationships, and forecast-horizon effects, conditioning the interpretation of other surveillance signals.

### 3.3 Models and data analysis procedure with simulation and empirical analyses

The data analysis framework integrates three complementary modeling paradigms to balance interpretability, flexibility, and predictive power. The first paradigm evaluates individual forecasting approaches independently, including LSTM, STL, and transformed-STL models. The second uses a mixed-effects framework to integrate heterogeneous predictors while accounting for spatial, demographic, and temporal variation. The third employs Transformer-based models with alternative spatial and temporal embeddings to capture complex spatiotemporal dependencies. Predictive performance was evaluated using standardized metrics, including Mean Absolute Error (MAE), Root Mean Squared Error (RMSE), Mean Absolute Percentage Error (MAPE), and Pearson correlation, where applicable. Model uncertainty was additionally assessed using confidence intervals (CI) or prediction intervals (PI), depending on the modeling approach.

• **Single Forecasting Approaches** The system followed a structured pipeline consisting of data preparation, forecasting, and evaluation. First, all datasets were cleaned to handle missing values and then split into training (70%) and testing (30%) subsets. Multiple forecasting approaches were evaluated independently, including Long Short-Term Memory (LSTM) [25], Seasonal-Trend Decomposition using Loess (STL) [41], and STL applied to preprocessed data. LSTM was used for its ability to capture sequential dependencies in time-series data, leveraging historical observations to improve prediction accuracy. The details of the LSTM hyperparameters used in this work are included in Table 2. STL decomposes time series into seasonal, trend, and residual components, providing a flexible approach for modeling temporal patterns and supporting weekly seasonality. The transformed-STL approach applied data transformations (e.g., log, square-root, moving average, or exponential smoothing) and scaling methods (standard, Min-Max, or robust scaling) prior to STL decomposition to improve model stability. Model evaluation was conducted using MAE, RMSE, Pearson correlation, and 95% CI, where applicable. MAE and RMSE were used to assess prediction error in absolute terms [42]. The Pearson correlation coefficient measures the strength and direction of the linear association between observed and predicted values [43]. Confidence intervals quantify uncertainty around estimated quantities and were reported at the 95% confidence level [44].
• **Mixed-Effects Model** Unlike the forecasting-oriented models in the previous section, which focus on capturing temporal dependencies through time-series decomposition and sequential learning, the mixed-effects model is designed to provide a transparent, interpretable representation of disease dynamics. While methods such as LSTM or STL emphasize predictive accuracy through statistical or deep learning techniques, the mixed-effects framework integrates heterogeneous predictors and explicitly models variation across counties, age groups, and temporal strata, making it particularly suited for explainability and stakeholder interpretation. We developed a transparent modeling process to enhance county-level pandemic prediction by integrating human clinical outcomes, wastewater surveillance, environmental conditions, and animal health within a non-linear mixed-effects framework. This modeling engine follows the OH-MEMA specification [33]. Fixed effects for multi-source predictors, random effects for spatial, temporal, and demographic grouping variables, and optional random slopes were all configured through an interactive interface and estimated by restricted maximum likelihood. The pipeline began with data cleaning, normalization, and STL decomposition to extract trends, seasonal components, and residuals. We incorporated polynomial terms to capture non-linearity [45]. For each county, continuous predictors (e.g., weather indicators) are log-transformed and expanded with cubic polynomial terms to capture non-linear relationships. This not only denoises predictors but also supports anomaly detection through residual-based thresholding with rolling statistics and confidence bands. For the selection of lags, we evaluated cross-correlations between the predictors and clinical outcomes across multiple counties. The chosen lags (−4 to 0 weeks) were selected based on maximizing correlation with the outcome while maintaining model stability. We also conducted a sensitivity analysis by varying the lag window (−6 to 2 weeks), which showed that predictive performance metrics (MAE, RMSE, correlation) were robust to reasonable variations in lag choice, confirming the appropriateness of the selected lag range. Additionally, we have expanded the structure of the random effects from a single grouping by county to a more comprehensive framework that considers variability by age group, sex group ratios, and temporal effects across different months. Model coefficients were estimated using restricted maximum likelihood (REML) in Python, with lag selection guided by maximum correlation and minimum RMSE. Coefficients for fixed effects were reported with 95% CI, and residual diagnostics (Q–Q plots and residual–fitted plots) [46] confirmed approximate normality and homoscedasticity. Prediction Intervals (PI) (80%–95%) were computed from residual distributions to assess uncertainty in weekly forecasts. The PI (80%–95%) represents the estimated range within which 80%–95% of future weekly case counts are expected to fall, based on model residual distributions, as was used by Bracher et al. (2021) [47]. Model evaluation was conducted using MAE, RMSE, Pearson correlation, CI, and PI. To assess whether differences between predictor configurations exceed sampling variation, we compared paired model configurations using a block bootstrap over weekly observations with 1000 resamples, which preserves the county and week structure. For each resample, we recomputed the correlation and error metrics for both configurations and retained the paired difference, from which we report the mean difference and its 95% percentile interval. The details of the hyperparameters of this model are included in Table 2. This approach enabled richer insight into pandemic dynamics while maintaining flexibility and user control in the modeling process.
• **Transformer-Based Spatiotemporal Modeling** We investigated the impact of spatiotemporal features on forecasting county-level COVID-19 incidence rates in the United States. We developed a learnable embedding for spatiotemporal features, which we combined with historical COVID-19 incidence rates to create a univariate time series feature. In our research, we utilized four types of embeddings for the COVID-19 data within a Transformer-based framework [16]. These embeddings included: no additional features, temporal embeddings, spatial embeddings, and spatiotemporal embeddings. For spatial embeddings, we combined US county-level population density [48] and the distance [49] from each county to its nearest neighbors as inputs. Specifically, we used the population density of the county along with the population densities of its three nearest neighbors, as well as the distances to these three neighbors. For temporal features, we used the day of the week, the day of the month, and the day of the year. The spatiotemporal embeddings combine both spatial and temporal features. In Fig. 2, at each step, we process the COVID-19 incidence rate along with temporal and spatial features relevant to the underlying county. These features are sent through an embedding layer that acts like a linear layer, mapping each type of feature to specific dimensions of the transformer model. This mapping corresponds to the internal dimensions used by the model. Once mapped, the features are combined using element-wise addition. The Transformer architecture consists of an encoder and a decoder. The combined embedded inputs are processed separately by both the encoder and decoder. During the decoding phase, these inputs are integrated into the cross-attention mechanism. After this integration, the final representations are sent through a fully connected layer. This layer’s size corresponds to the number of steps for the forecast that the model is making. To evaluate the effectiveness of these embeddings, we performed an ablation study by training separate models to forecast the COVID-19 incidence rate for 7, 14, and 21 days. The details of Transformer’s experimental setup and hyperparameters are included in Table 2. The dataset was sourced from daily cumulative COVID-19 confirmed cases reported by The New York Times [50] and included data from 3204 counties across the United States, covering the period from March 2020 to March 2021. This timeframe included significant spikes before the vaccine rollout. For the analysis, we used eight months of data (March to October 2020) for training, two months (November to December 2020) for validation, and three months (January to March 2021) for testing. Although the training dataset included all 3204 counties, a subset of these counties reported insufficient data during the testing period. Consequently, the test data included a total of 3042 counties. For each county and data split, input–output sequences were generated sequentially. Specifically, we began with 20 days of historical data as input and predicted the subsequent 7-, 14-, and 21-day horizons. For subsequent sequences within the same county and split, the input window was extended by one day rather than using a fixed-length moving window. This approach allowed the models to incorporate the entire available history of incidence-rate data up to each prediction point. The corresponding future sequences were then used as prediction targets, and this process was repeated until the end of each split period was reached. We compared the performance of models without feature embeddings with models using different types of embeddings. Additionally, we compared the Transformer-based models to the autoregressive integrated moving average (ARIMA) model, which serves as our baseline. The parameters of the ARIMA model—specifically, the order of the autoregressive component p, the degree of differencing d, and the order of the moving average component q —were determined using an automated search method. This method utilized a stepwise approach to find the best combination of (p,d,q) values within the default ranges: p and q from 2 to 5, and d from 1 to 2, while minimizing the Akaike Information Criterion (AIC). Because the ARIMA model is a statistical approach, this parameter search was applied consistently across all time series sequences generated for each county and for forecasting horizons. For each time series sequence, the model producing the lowest AIC was selected. The corresponding forecasts for each sequence were then used to evaluate overall model performance. The model was fitted in a non-seasonal configuration, as the available time series spanned only 13 months, which was insufficient to capture reliable seasonal patterns. The details of ARIMA’s hyperparameters are included in Table 2.

**Visualization and Interactive Analysis**

**Fig. 2:**
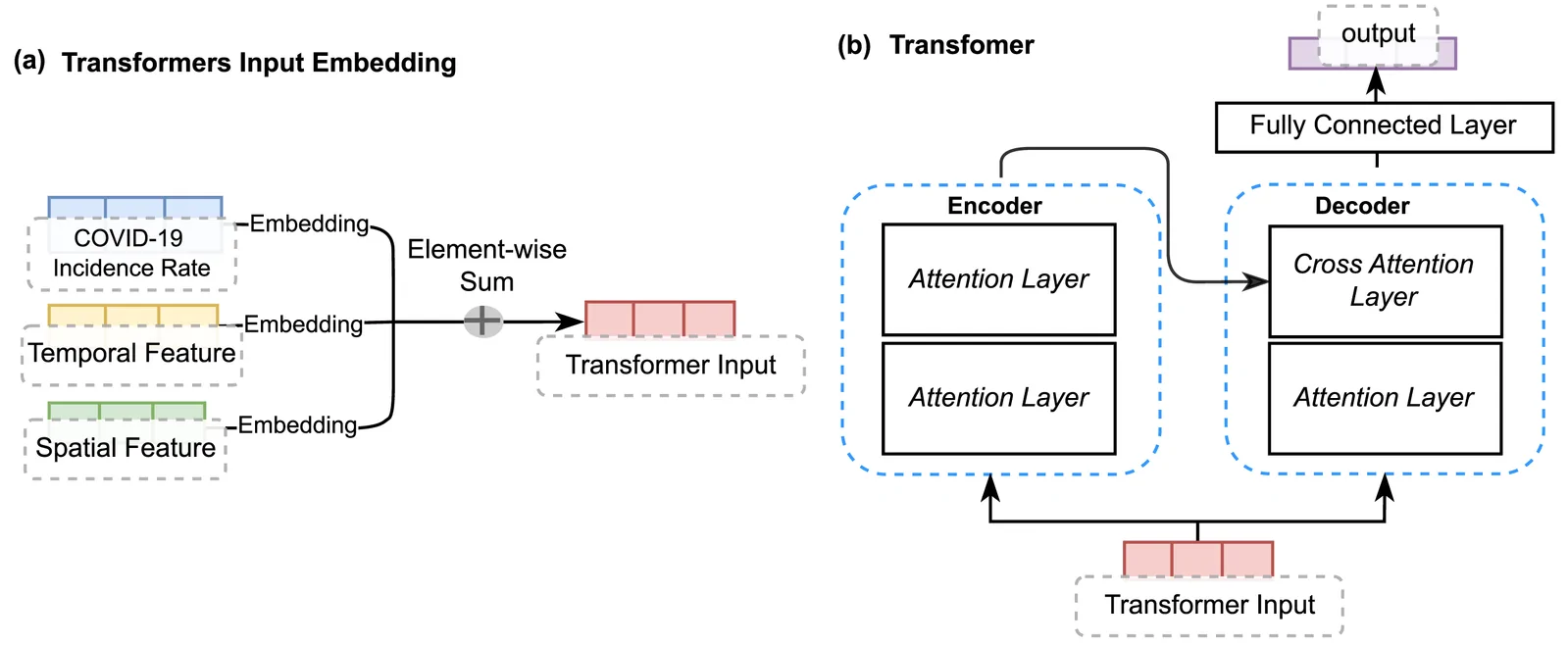
(a) Transformers Input Embedding: spatial and temporal features, along with COVID-19 incidence rates, are fed into the embedding layer, which integrates them as input for the Transformer architecture. (b) Transformer Architecture: this model processes the combined input to generate forecasts for 7, 14, and 21 days in a single run.

**Table 2:** Hyperparameter tuning ranges, selection criteria, and best values for each model.

Model	Hyperparameter	Range/Values tested	Selection criteria	Best value (Example)
ARIMA	p	[2–5]	Lowest AIC for a given time-series	Differs for each time-series
	q	[2–5]	Lowest AIC for a given time-series	Differs for each time-series
	d	[1–2]	Lowest AIC for a given time-series	Differs for each time-series

Transformer	Model Dimension	[64, 128, 256, 512]	Lowest MAPE/RMSE	64
	Attention Heads	[2, 4, 8]	Lowest MAPE/RMSE	2
	Batch Size	[8, 16, 32, 64, 128, 256]	Lowest MAPE/RMSE	16
	Epochs	20	Validation early stopping (no improvement after 5 epochs)	20
	Feed Forward Size	[512, 1024]	Lowest MAPE/RMSE	1024
	Dropout	[0.05, 0.1, 0.15]	Lowest MAPE/RMSE	0.1

LSTM	Hidden Units	[32, 64, 128, 256]	Lowest validation loss (MAE)	64
	Batch Size	[16, 32, 64]	Best validation MAE	32
	Number of Layers	[1–4]	Captures temporal hierarchy and long-term dependencies	3
	Epochs	[50–300]	Early stopping (no improvement after 10 epochs)	50
	Optimizer	Adam/RMSprop	Validation loss	Adam
	Sequence Length	[2, 4, 8, 12 weeks]	Lag sensitivity	2 weeks

Mixed-Effects	Fixed Effects	Wastewater, NET, Animal predictors	Lower MAE/RMSE	Wastewater + NET + Animal
	Random Effects	County, Month, Age, Sex	Lower MAE/RMSE	County + Month
	Lag Values	[−6 to 2 weeks]	Higher Spearman correlation and Lower MAE/RMSE	4 weeks ahead
	Polynomial Degree	[1–4]	$R^{2}$ improvement	3
	Transformations	Log-transform, STL decomposition	Lower MAE/RMSE	Log + STL
	Smoothing	None/STL	Residual diagnostics	STL

The visualization framework provides an interactive environment for exploring, analyzing, and interpreting multi-domain One Health data in a transparent and user-friendly manner. Central to this framework is the Predictive Intelligence for Pandemic Prevention (PIPP) dashboard, as shown in Fig. 3, which integrates heterogeneous data streams including human clinical outcomes, wastewater surveillance, and environmental indicators across spatial and temporal scales. Through layered visualizations such as choropleth maps, linked time-series charts, sunburst diagrams, and lag-aware heatmaps, users can examine lead–lag relationships, temporal trends, and regional differences. On the other hand, the mixed-effects model tool, as shown in Fig. 4, provides a transparent predictive framework that allows users to configure fixed and random effects, apply lagged and polynomial expansions, and interactively explore model outputs. Together, these tools combine advanced analytics with intuitive visual exploration, enabling stakeholders to detect anomalies, validate predictive models, and gain actionable insights into the complex dynamics of infectious diseases under the One Health paradigm.

**Fig. 3:**
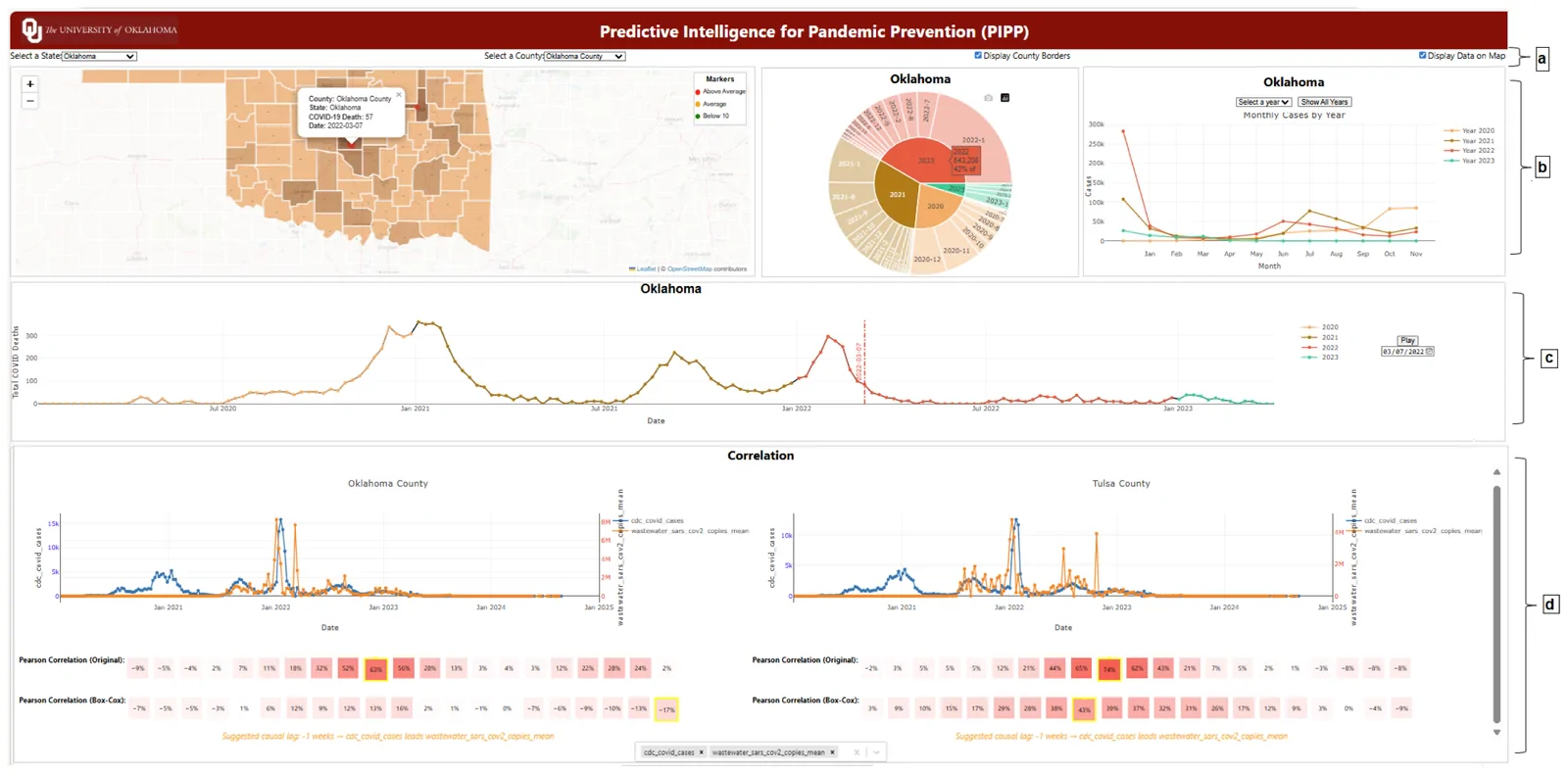
Predictive Intelligence for Pandemic Prevention (PIPP) Dashboard. The dashboard views include (a) Options Panel to select a region of interest (state/county); (b) three types of interactive visualizations: An interactive map exploring disease spread in the region using a tooltip, a Sunburst diagram, and line charts to visualize the data hierarchically; (c) a line chart that displays COVID-19 deaths or cases for the whole state with an automatic timer that will go through the line chart to display the information for every week, and at the same time, it changes the details on the interactive map for each county; (d) Combined Datasets: Each selected county has a line chart with two different datasets and a heatmap to visualize the correlation.

• **PIPP Dashboard** The PIPP dashboard (Fig. 3) is an interactive, web-based platform designed to support pandemic surveillance through an integrative One Health approach [51]. It combines multiple heterogeneous data streams, including clinical cases, hospitalizations, wastewater monitoring, and weather factors, into a unified system for exploration and analysis for the years 2020–2023 in Oklahoma at the county level. The dashboard offers layered visualizations that enable users to examine both spatial and temporal dimensions of disease dynamics.
  Key features include a choropleth map for normalized regional comparisons, temporospatial visualizations that link maps with time-series charts, and interactive brushing and focus+context techniques for detailed temporal exploration. Comparative analysis is supported through dual-axis line charts and multi-series panels, enabling users to examine relationships across regions and datasets simultaneously.
  To further enhance exploratory analysis, we modified the dashboard to include lag-aware correlation heatmaps that characterize temporal lead–lag associations between two selected data series. Two heatmaps are provided: one for the original Pearson correlations and another for Box–Cox-transformed correlations over a user-defined lag range (e.g., -10 to +10 weeks), as shown in Fig. 3. This design enables users to examine whether changes in one variable consistently precede or follow changes in another and to identify potential early-warning signals in pandemic surveillance. Interactive features allow users to hover over each heatmap cell to inspect the correlation value at a given lag and explore potential temporal associations between the two time series. These lagged correlations are used for exploratory and predictive interpretation and are not interpreted as evidence of causality.
  Built with Python for data management and React + Recharts for visualization, the dashboard is scalable, user-friendly, and accessible via the web. By unifying diverse datasets and embedding interactive analytics, it facilitates early detection, comparative analysis, and a richer understanding of the complex factors influencing pandemic spread and impact.
• **Mixed-Effects Model Tool** The mixed-effects modeling tool complements the dashboard by providing a transparent and flexible predictive framework for county-level pandemic outcomes, as shown in Fig. 4. It integrates One Health data sources, including human clinical outcomes, wastewater surveillance, and environmental indicators, within a non-linear mixed-effects model to account for both fixed and random effects.
  The modeling pipeline begins with rigorous data preparation, including cleaning, normalization, and layered STL decomposition to extract smoothed trends, seasonal components, and anomalies. Residual-based anomaly detection with confidence bands supports the identification of potential outbreaks or unusual patterns. Predictors are log-transformed, expanded with polynomial terms, and systematically lagged to capture lead–lag effects, ensuring both non-linear and temporal relationships are represented.
  The tool is integrated into a visual interface that allows users to interactively choose outcomes, predictors, lag windows, and random effect structures. This interactive design supports exploratory modeling, transparency in feature selection, and the identification of key relationships, offering an adaptive framework for predictive pandemic intelligence. The full system is deployed and freely accessible via a web interface^1^ .Its architecture, interaction design, provenance model, and a formative expert evaluation are reported in detail in our companion paper on OH-MEMA [33]. Users can utilize the provided sample dataset or upload their own dataset to view the results quickly and easily. Our framework integrates epidemiological modeling with visual analytics, enabling clear and data-driven decision-making in public health. This work highlights the potential of a One Health visual analytics approach for predicting emerging threats, informing interventions, and enhancing resilience against future pandemics.

**Fig. 4:**
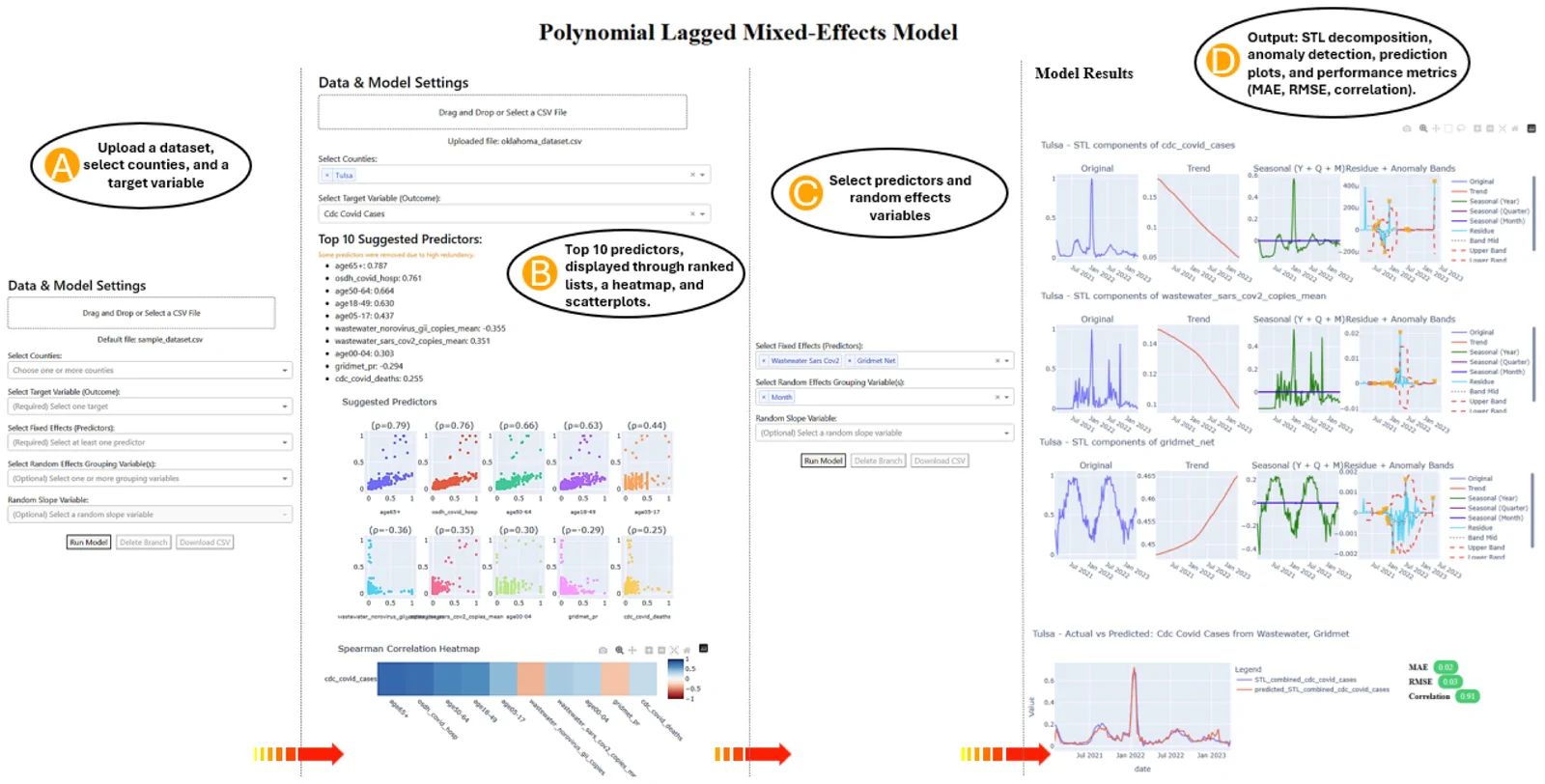
Visual Analytics Workflow for Polynomial Lagged Mixed-Effects Modeling. (A) After uploading a dataset, users select counties and a target variable. (B) The system calculates Spearman correlations between the target and all other variables, displaying the top 10 predictors via ranked lists, a heatmap, and scatter plots. (C) Users configure fixed and random effects, optionally specify interaction (slope) variables, and run the model. (D) Output includes STL decomposition, anomaly detection, prediction plots, and model performance metrics (MAE, RMSE, correlation).

## 4 Results

### 4.1 Cross-case validation strategy

The case studies were designed to evaluate complementary aspects of the proposed One Health modeling framework rather than to represent identical forecasting tasks. Accordingly, validation focused on whether the framework could (i) capture temporal heterogeneity in disease outcomes, (ii) generalize this modeling strategy across different outcomes and geographic settings, (iii) evaluate the incremental contribution of heterogeneous One Health surveillance signals, and (iv) incorporate spatial and temporal context across different forecast horizons.

The mixed-effects analyses use internal model comparison, in which alternative predictor sets and random-effects structures are evaluated against one another under identical data and validation conditions, whereas the Transformer analysis is compared against an ARIMA statistical baseline appropriate to univariate sequence forecasting. The two comparison strategies answer different questions and are therefore reported separately rather than merged into the single ranking.

The Arizona and Arkansas analyses evaluate the contribution of temporal random effects using environmental predictors and different respiratory-disease outcomes. The Oklahoma analysis extends this evaluation to a richer One Health setting by combining wastewater, environmental, and animal-health signals and comparing alternative random-effects structures and preprocessing configurations. The Transformer experiment provides a complementary evaluation of spatial and temporal representations for 7-, 14-, and 21-day forecasts. Across the case studies, predictive performance was assessed using error and association metrics appropriate to each modeling task.

Uncertainty was quantified using confidence and prediction intervals for the Arizona and Arkansas analyses, using repeated-configuration comparison for the Oklahoma analysis, and using variability estimates across counties for the Transformer forecasts. Interval estimates are therefore reported wherever the modeling task supports a directly comparable interval construction.

• **Single Prediction Model Results** In our previous study [51], we conducted a county-level analysis of weekly COVID-19 cases and deaths in Oklahoma for the years 2020–2022, employing various forecasting methodologies to enhance predictive accuracy. We found that the correlation coefficients ranged from 0.816 to 0.916, indicating a consistently strong linear relationship between predicted and observed values. Among the transformation-based methods, the Log + STL approach demonstrated the highest correlation, followed by the SQRT + STL approach. In terms of error metrics, LSTM and RobustScaler + STL achieved the lowest MAE and RMSE, reflecting superior predictive accuracy. Furthermore, methods based on STL decomposition combined with scaling exhibited the narrowest confidence intervals, signifying greater precision and reliability in the forecasts.
• **Mixed-Effects Model Results** For the following case studies, we selected the two most populous counties in each state and integrated environmental, clinical, and wastewater data sources into mixed-effects models to evaluate their predictive power for respiratory-related outcomes. In our case studies, we calculated the weekly mean NET values from the gridMET meteorological data for each county, based on Eq. (1), which serves as a key environmental predictor of respiratory-related health outcomes.
  Each case study is supported by a table of the results and an analytics provenance tree. Each tree branch represents a unique modeling configuration, including the selected target variable, predictors, random effects, and corresponding model performance metrics. Leaf nodes display correlation (ρ), MAE, and RMSE. Node colors provide quick insight into model quality: green indicates strong correlation (ρ>0.7) or low error (MAE/RMSE <0.1), while red highlights weaker performance. Additionally, node size encodes the magnitude of performance; larger nodes correspond to stronger correlations or lower errors, helping users identify optimal models at a glance.
  – **Case Study I: Arizona (Flu and RSV Cases)** For Arizona, we modeled weekly county-level influenza and RSV cases using the Net Effective Temperature (NET) as the primary environmental predictor. Models were evaluated both with and without the month included as a random effect to capture temporal variability. When investigating across all Arizona counties, the models showed a consistent pattern: average correlations increased by approximately 10%–15% when month was included as a random effect, while MAE and RMSE decreased accordingly. On average, RSV models achieved statewide correlations of approximately 82% with month effects (compared to 76% without), and flu models reached around 79% (versus 71%). For example, Table 3 summarizes model performance for Maricopa and Pima counties, while Fig. A.5 - Appendix, provides a visual provenance view of modeling decisions. Results indicate that incorporating the month as a random effect improved predictive performance across both counties, particularly for RSV. For example, in Maricopa County, RSV predictions achieved a correlation of 89% with month effects compared to 84% without. These results highlight the value of accounting for seasonal variation in respiratory disease dynamics.
    This demonstrates that while performance varies between individual counties, the overall statewide trends confirm the robustness of including temporal structure in the mixed-effects framework. The Arizona analysis therefore provides the first validation step for the framework: incorporating temporal heterogeneity through a month-level random effect improved predictive performance relative to models without this effect. The result was observed for both influenza and RSV, although the magnitude of improvement differed between outcomes. This suggests that environmental predictors such as NET should be interpreted within an appropriate temporal structure rather than treated as uniformly informative across weeks and seasons.
  – **Case Study II: Arkansas (COVID-19 Cases and Deaths)** For Arkansas, weekly county-level COVID-19 cases and deaths were modeled using the Net Effective Temperature (NET) as the primary environmental predictor. Models were evaluated both with and without the month included as a random effect to account for temporal variability.
    When averaging across all Arkansas counties, we observed a consistent improvement when incorporating the month as a random effect. On average, statewide correlations for case predictions increased from about 53% without month effects to 80% with them, while death predictions rose from roughly 38% to 72%. Correspondingly, MAE and RMSE values decreased, underscoring the value of including temporal variation, as shown in Table 4. These statewide results confirm the patterns observed in Pulaski and Washington counties, where accounting for monthly variability substantially enhanced the predictive accuracy of NET-based models for both COVID-19 cases and deaths. Table 4 presents the results for Pulaski and Washington counties, complemented by the provenance visualization in Fig. A.6 - Appendix. In Pulaski County, the correlation for COVID-19 cases increased from 43% to 79%, while deaths improved from 31% to 65%. A similar pattern was observed in Washington County, where case prediction improved from 54% to 78% and death prediction from 36% to 71%. Although death prediction showed weaker correlations compared to cases, reflecting noisier and less frequent reporting, the inclusion of month as a random effect substantially enhanced overall model accuracy. The Arkansas analysis provides a second validation of the temporal-heterogeneity component using different outcomes from the Arizona case study. The improvement was observed for both COVID-19 cases and deaths, although the magnitude differed substantially between the two outcomes. This finding indicates that the benefit of hierarchical temporal structure is not restricted to a single respiratory disease or outcome type, while also demonstrating that model performance remains outcome-dependent.
  – **Case Study III: Oklahoma (COVID-19 Hospitalizations)** For Oklahoma, we evaluated whether heterogeneous surveillance signals from human, environmental, wastewater, and animal health domains could jointly improve prediction of weekly COVID-19 hospitalizations. The predictor set included wastewater viral concentrations (SARS-CoV-2, influenza, and RSV), Net Effective Temperature (NET), and aggregated canine and feline veterinary activity. Wastewater measurements provide a community-level pathogen signal, NET provides environmental context, and veterinary activity provides an additional syndromic signal from the companion-animal domain. The animal dataset reports the number of distinct veterinary visits per county per week and therefore reflects patterns of companion animal clinical encounters. We use these signals to test whether animal health surveillance contains predictive information that complements wastewater and environmental measurements when forecasting human hospitalizations. Models were evaluated under different combinations of predictors and random effects, including county, month, age group, and sex group, with and without STL preprocessing. Across all Oklahoma counties, mixed-effects models that incorporated month and age group as random effects consistently demonstrated strong performance. On average, correlations reached approximately 70%–80%, with MAE around 0.11 and RMSE around 0.15. Applying STL decomposition further improved predictive accuracy, lowering the MAE to as little as 0.053, the RMSE to 0.085, and increasing the correlation to over 91%. These results indicate that combining wastewater, environmental, and animal syndromic predictors can provide complementary predictive information for forecasting COVID-19 hospitalizations in the evaluated setting. Focusing specifically on Tulsa County, wastewater and NET were used as fixed effects, while random effects for county, month, age group, and sex group captured demographic and temporal heterogeneity. STL preprocessing was also applied to assess its impact on predictive performance. Table 5 shows the predictive performance of models for weekly COVID-19 hospitalizations in Tulsa County, Oklahoma. Models combined different One Health predictors—wastewater viral concentrations, NET, and animal health data—with various random effects (county, month, age group, and sex group), and were evaluated with and without STL preprocessing. Results demonstrate that STL substantially improved predictive accuracy, yielding consistently lower MAE and RMSE and higher correlations. Among all configurations, Model M11 (Wastewater + NET + Animal (Canine)) with county, month, and age group as random effects achieved an MAE of 0.0531, RMSE of 0.0847, and correlation of 91.86%. The model comparisons provide a more direct assessment of the contribution of the animal health signal. Models using wastewater alone or NET alone provided lower predictive performance than models combining wastewater and environmental information. For example, under the county + month + age-group random-effects structure, the wastewater + NET model (M3) achieved a correlation of 89.47% without STL and 89.47% with STL, whereas the corresponding full models incorporating animal-health information achieved correlations of 88.60% for feline data (M10) and 91.86% for canine data (M11) after STL. The canine-containing configuration therefore produced the strongest overall performance among the evaluated models, with an MAE of 0.0531 and RMSE of 0.0847. These results suggest that canine syndromic information can provide complementary predictive information in this setting; however, the results should not be interpreted as evidence that animal respiratory illness causes or directly transmits human COVID-19. Rather, the animal variables represent an additional surveillance signal whose predictive utility can be evaluated within the integrated modeling framework. These results indicate that predictive performance depends on both the composition of the surveillance signals and the representation of temporal and demographic heterogeneity. In particular, the strongest configuration combined wastewater, NET, and canine syndromic information with county, month, and age-group random effects after STL preprocessing. This finding supports the value of evaluating animal-health information as a complementary surveillance signal rather than assuming that all One Health data sources contribute equally across outcomes.
• **Transformers with Spatio-Temporal Embeddings Results** The results in Table 6 illustrate the averaged performance of the Transformer model and ARIMA across the generated sequences for three forecast horizons—7, 14, and 21 days—calculated for all counties in the US. This evaluation focused on the test set during the period from January to March 2021. We evaluate the performance of our models using two key error metrics: RMSE and MAPE. We explored four different feature embeddings for the Transformer architecture: (1) no additional features, (2) temporal features, (3) spatial features, and (4) a combination of both. For each forecast horizon, we counted the frequency of instances where either the MAPE or RMSE achieved the lowest value. Based on our findings, the results indicate that the Transformer model’s version utilizing spatial features outperforms the others in the 21-day forecast horizon. Meanwhile, the combination of spatial and temporal features yields the best results for the 7- and 14-day forecast horizons. This highlights the advantage of incorporating spatial and spatio-temporal features compared to not using any additional features. In addition, we conducted a county-level error analysis for all forecast horizons by comparing the use of both spatio-temporal features and the performance without additional features in the Transformer model. Table 7 shows the improvement rates for three forecast horizons across all counties. The Transformer model shows that 98.3%, 97.6%, and 94.9% of counties have improved performance across these horizons. This demonstrates the advantage of using both features across all horizons compared to no additional feature embedding. The Transformer experiment provides a complementary validation of contextual representation across forecast horizons. At 7 and 14 days, the combined spatial–temporal embedding achieved the lowest MAPE and RMSE, whereas spatial features alone performed best at 21 days. The results therefore indicate that the predictive contribution of contextual information varies with forecast horizon. Compared with the ARIMA baseline, all evaluated Transformer configurations achieved lower MAPE and RMSE, with the largest performance gains obtained using spatial–temporal representations. These results complement the mixed-effects analyses by demonstrating that the framework can evaluate both interpretable hierarchical models and nonlinear spatiotemporal forecasting architectures under different prediction horizons.

**Table 3:** Model performance for Flu and RSV cases prediction for the State of Arizona, Maricopa and Pima counties, using NET with and without random effects (Month).

County	Target	Predictors	Random effects	Corr. (%)	95% CI	MAE	RMSE	PI (80%–95%)
Maricopa	Flu	NET	Month	80	[76, 84]	4.45	7.10	[–0.3, 13.5]
			–	77	[72, 81]	6.00	9.30	[–0.6, 15.0]
	RSV	NET	Month	89	[86, 92]	3.10	5.20	[–0.2, 10.0]
			–	84	[80, 88]	4.70	7.50	[–0.4, 12.5]

Pima	Flu	NET	Month	78	[74, 82]	5.90	9.20	[–0.4, 17.5]
			–	77	[72, 81]	6.20	9.50	[–0.5, 18.0]
	RSV	NET	Month	84	[80, 87]	6.20	9.80	[–0.3, 16.0]
			–	81	[77, 85]	7.10	10.8	[–0.4, 18.5]

State Average	Flu	NET	Month	79	[75, 83]	5.20	8.20	[–0.3, 15.0]
			–	71	[66, 76]	6.10	9.40	[–0.5, 17.0]
	RSV	NET	Month	82	[78, 86]	5.60	9.20	[–0.2, 16.5]
			–	76	[71, 80]	7.60	11.3	[–0.4, 18.5]

**Table 4:** Model performance for COVID-19 prediction for the State of Arkansas, Pulaski and Washington counties, using NET with and without random effects (Month).

County	Target	Predictors	Random effects	Corr. (%)	95% CI	MAE	RMSE	PIs (80%–95%)
Pulaski	Cases	NET	Month	79	[75, 83]	3.91	6.50	[−0.3, 13.1]
			–	43	[38, 48]	4.40	7.60	[−0.6, 14.7]
	Deaths	NET	Month	65	[60, 70]	0.12	0.20	[0, 0.48]
			–	31	[26, 36]	1.13	1.47	[0, 3.3]

Washington	Cases	NET	Month	78	[74, 82]	1.89	3.32	[−0.2, 6.0]
			–	54	[49, 59]	2.69	4.23	[−0.3, 7.7]
	Deaths	NET	Month	71	[66, 76]	0.10	0.14	[0, 0.32]
			–	36	[31, 41]	1.13	1.36	[0, 3.4]

State Average	Cases	NET	Month	80	[76, 84]	2.90	5.14	[−0.2, 9.6]
			–	53	[48, 58]	3.82	6.14	[−0.5, 12.8]
	Deaths	NET	Month	72	[67, 77]	0.11	0.27	[0, 0.62]
			–	38	[33, 43]	1.10	1.34	[0, 3.2]

**Table 5:** Performance of prediction models for COVID-19 hospitalizations in Tulsa County, Oklahoma, including Wastewater, NET, and Animal (Feline and Canine) predictors with and without STL decomposition. Best values per model are in **bold**.

Model	Random effects	MAE	RMSE	Corr. (%)	MAE (STL)	RMSE (STL)	Corr. (STL) (%)
M1: Wastewater	County	0.1399	0.1977	63.71	0.0899	0.1377	66.71
	Month	0.1196	0.1729	75.92	0.0696	0.1029	85.12
	Sex Group	0.1297	0.1955	65.79	0.0927	0.1365	67.79
	Age Group	0.1213	0.1860	70.91	0.0913	0.1360	67.91
	C+M+S	0.1102	0.1496	78.21	0.0802	0.1178	85.32
	C+M+A	0.0827	0.1073	82.50	0.0627	0.1003	86.50

M2: NET	County	0.1802	0.2478	52.32	0.1002	0.1478	66.32
	Month	0.1362	0.1907	66.60	0.0762	0.1007	86.20
	Sex Group	0.1802	0.2478	52.32	0.1151	0.1489	62.32
	Age Group	0.1802	0.2478	52.32	0.1102	0.1478	62.62
	C+M+S	0.1370	0.1908	66.46	0.0802	0.1196	83.21
	C+M+A	0.1370	0.1908	66.46	0.0802	0.1178	85.32

M3: Wastewater + NET	County	0.1208	0.1802	74.21	0.0808	0.1202	77.21
	Month	0.0856	0.1089	84.35	0.0696	0.0989	88.35
	Sex Group	0.1159	0.1777	77.10	0.0807	0.1201	77.10
	Age Group	0.1084	0.1597	79.03	0.0804	0.1137	79.03
	C+M+S	0.0835	0.1084	85.69	0.0735	0.1084	86.69
	C+M+A	0.0673	0.0930	89.47	0.0673	0.0930	89.47

M4: Animal (Feline)	County	0.1931	0.2623	27.46	0.1936	0.2618	30.46
	Month	0.1326	0.1895	70.41	0.0926	0.1195	81.41
	Sex Group	0.1932	0.2624	27.36	0.1936	0.2618	30.46
	Age Group	0.1932	0.2624	27.29	0.1936	0.2618	30.46
	C+M+S	0.1397	0.1948	68.21	0.0897	0.1148	82.21
	C+M+A	0.1397	0.1948	68.21	0.0886	0.1124	82.43

M5: Animal (Canine)	County	0.1936	0.2618	30.08	0.1136	0.1518	37.08
	Month	0.1314	0.1897	70.29	0.0814	0.1197	83.29
	Sex Group	0.1936	0.2618	30.10	0.1136	0.1518	37.08
	Age Group	0.1936	0.2618	30.08	0.1136	0.1518	37.08
	C+M+S	0.1388	0.1942	69.50	0.0835	0.1084	85.69
	C+M+A	0.1388	0.1942	69.50	0.0830	0.1068	85.74

M6: Wastewater + Animal (Feline)	County	0.1319	0.1982	66.11	0.0919	0.1382	68.11
	Month	0.0937	0.1335	83.00	0.0637	0.0935	88.00
	Sex Group	0.1319	0.1982	66.13	0.0919	0.1382	66.13
	Age Group	0.1202	0.1732	76.11	0.0902	0.1132	76.11
	C+M+S	0.0864	0.1243	83.38	0.0864	0.1243	83.38
	C+M+A	0.0682	0.0968	88.37	0.0682	0.0968	88.37

M7: Wastewater + Animal (Canine)	County	0.1314	0.1973	66.68	0.0914	0.1373	68.68
	Month	0.0900	0.1304	83.85	0.0700	0.0904	87.85
	Sex Group	0.1314	0.1973	66.69	0.1314	0.1973	66.69
	Age Group	0.1142	0.1726	77.68	0.1142	0.1726	77.68
	C+M+S	0.0854	0.1163	84.53	0.0854	0.1163	84.53
	C+M+A	0.0674	0.0941	89.52	0.0674	0.0941	89.52

M8: NET + Animal (Feline)	County	0.1811	0.2482	53.01	0.1011	0.1482	64.01
	Month	0.1298	0.1842	71.40	0.0798	0.1042	86.40
	Sex Group	0.1811	0.2482	53.01	0.1011	0.1482	64.01
	Age Group	0.1621	0.2182	63.01	0.1011	0.1480	65.01
	C+M+S	0.1287	0.1833	72.05	0.0845	0.1121	84.05
	C+M+A	0.1245	0.1721	78.05	0.0745	0.1021	85.05

M9: NET + Animal (Canine)	County	0.1805	0.2451	56.22	0.0905	0.1351	70.22
	Month	0.1296	0.1844	71.31	0.0796	0.1044	84.31
	Sex Group	0.1805	0.2451	56.23	0.1305	0.1951	66.23
	Age Group	0.1282	0.1813	67.22	0.1282	0.1813	67.22
	C+M+S	0.1276	0.1829	72.25	0.0776	0.1029	84.45
	C+M+A	0.1239	0.1719	78.31	0.0739	0.1019	85.31

M10: Wastewater + NET + Animal (Feline)	County	0.1292	0.1820	65.00	0.0892	0.1220	79.00
	Month	0.0934	0.1333	83.05	0.0634	0.0833	90.05
	Sex Group	0.1292	0.1820	65.01	0.1091	0.1482	78.62
	Age Group	0.1196	0.1729	75.92	0.0882	0.1210	79.92
	C+M+S	0.1091	0.1482	78.62	0.0791	0.0982	86.62
	C+M+A	0.0736	0.1023	83.60	0.0736	0.0923	88.60

M11: Wastewater + NET + Animal (Canine)	County	0.1274	0.1750	68.34	0.0874	0.1150	76.34
	Month	0.0735	0.0984	86.93	0.0635	0.0984	87.93
	Sex Group	0.1274	0.1750	68.35	0.0874	0.1150	76.35
	Age Group	0.1115	0.1611	79.34	0.0815	0.1011	79.34
	C+M+S	0.0667	0.0923	87.87	0.0567	0.0823	89.87
	C+M+A	0.0731	0.0921	88.86	**0.0531**	**0.0847**	**91.86**

C: County, M: Month, S: Sex group, A: Age group.

**Table 6:**
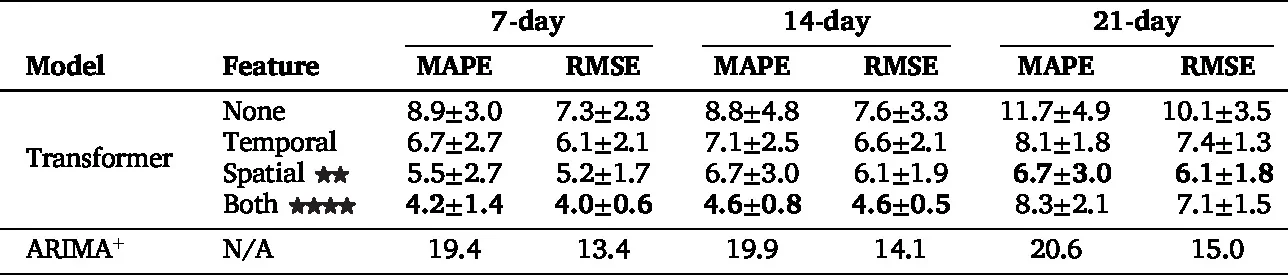
Evaluation of incidence rate forecasting across various horizons, for all counties in the US, comparing MAPE and RMSE for Transformer and ARIMA (statistical baseline). The Transformer was tested with four feature embeddings: none, temporal, spatial, and combined. Results show that adding spatial and spatio-temporal features improves Transformer performance.

Number of times one feature embedding outperforms others across three forecast horizons.

+ ARIMA: Autoregressive Integrated Moving Average.

**Table 7:** Comparison of county-level forecast changes using both features versus without additional features across time horizons. The table details the number of counties where Transformer demonstrated improved or worsened performance, and the rate of improvement to total changes.

Model	7-day			14-day			21-day		
	Improved	Worsened	Impr. rate %	Improved	Worsened	Impr. rate %	Improved	Worsened	Impr. rate %
Transformer	2991	51	**98.3**	2970	72	**97.6**	2888	154	**94.9**

## 5 Discussion

This study presents a comprehensive One Health visual analytics framework that integrates clinical, environmental, wastewater, and animal-health data to improve syndromic surveillance and forecasting of respiratory-related outcomes. Our analyses show that combining multiple surveillance domains within mixed-effects models improves predictive performance for most configurations, and that the framework makes the contribution of each domain explicit rather than assumed. Table 8 synthesizes the case studies by linking each one to its data sources, modeling and validation strategy, principal finding, and practical One Health implication, so that readers can assess the effect of modeling choices such as preprocessing, hierarchical random effects, and predictor selection at a glance.

Taken together, the case studies demonstrate complementary validation of the framework rather than a single uniform forecasting benchmark. The Arizona and Arkansas analyses show that incorporating temporal heterogeneity through random effects can substantially improve predictive performance across different respiratory outcomes. The Oklahoma analysis extends this result by evaluating heterogeneous surveillance domains and demonstrates that the strongest-performing configuration combined wastewater, environmental, and canine syndromic information with hierarchical random effects and STL preprocessing. The Transformer analysis further demonstrates that the usefulness of contextual information depends on the forecast horizon, with combined spatial–temporal embeddings performing best for 7- and 14-day forecasts and spatial embeddings performing best at 21 days. Thus, the case studies collectively support the framework’s ability to compare data contributions, model structures, and contextual representations rather than establishing that a single model or data source is universally superior.

**Table 8:** Cross-case summary of data sources, modeling approaches, validation strategies, key findings, and One Health implications.

Case Study	Data Sources	Modeling and Validation	Key Finding	One Health Implication
**Arizona**	Human flu/RSV outcomes + NET	Nonlinear mixed-effects; compared models with and without month random effects using MAE, RMSE, correlation, and CI/PI.	Month effects improved correlation and reduced MAE/RMSE, with strongest gains for RSV.	Environmental predictors require temporal context to represent seasonal heterogeneity.

**Arkansas**	Human COVID-19 cases/deaths + NET	Nonlinear mixed-effects; compared models with and without month random effects using MAE, RMSE, correlation, and CI/PI.	Case correlation increased from 53% to 80%; death correlation from 38% to 72% with temporal effects.	Hierarchical temporal structure improves prediction across heterogeneous outcomes.

**Oklahoma**	Human hospitalizations + wastewater + NET + canine/feline syndromic data	Nonlinear mixed-effects; compared predictor combinations, random-effects structures, and STL preprocessing using MAE, RMSE, and correlation	Best configuration (wastewater + NET + canine; county + month + age; STL) achieved MAE 0.0531, RMSE 0.0847, and correlation 91.86%.	Multiple surveillance domains can provide complementary predictive information; animal-health utility is species- and configuration-dependent.

**U.S. Transformer**	County COVID-19 incidence + spatial/temporal features	Transformer; compared no, temporal, spatial, and combined embeddings across 7/14/21-day horizons using MAPE and RMSE.	Combined spatial–temporal features performed best at 7/14 days; spatial features performed best at 21 days and outperformed ARIMA.	Forecasting performance depends on spatial and temporal context and forecast horizon.

While prior studies have applied mixed-effects modeling or deep learning to a single dataset, our framework unifies multiple One Health data streams in an interactive and interpretable platform. Although the majority of model predictions were consistent with expectations, we observed lower correlations for death predictions in certain counties, likely due to sporadic reporting and low event counts in these areas. This highlights the importance of accounting for data quality and reporting frequency when interpreting model outputs.

The reliability and generalizability of the framework depend heavily on data availability and consistency across domains. Wastewater-based surveillance, for instance, requires standardized sampling schedules, processing protocols, and quantification methods, which are not uniformly implemented across all regions. Similarly, while NET serves as an efficient composite weather indicator, it may not fully represent localized meteorological variations in areas with microclimates or sparse station coverage. To mitigate these limitations, the framework supports flexible model specification and cross-validation across spatial and temporal subsets, enabling users to assess robustness under varying data conditions.

When discussing animal health data, it is important to note that the companion animal health information used in this study comes exclusively from Banfield Pet Hospital. Therefore, this data represents only a subset of the larger pet population and may be affected by factors such as geographic distribution, socioeconomic status, and healthcare-seeking behaviors of Banfield clients. This sampling bias could result in the underrepresentation of animals that do not regularly visit veterinary clinics or those attended by other providers. This reflects both the proprietary nature of the dataset and broader challenges in accessing comprehensive veterinary health data, as such information is not publicly available and often restricted due to privacy and ownership constraints.

The Oklahoma results also suggest that the contribution of animal health data is species- and configuration-dependent. Canine syndromic information produced the strongest performance among the evaluated animal health configurations, whereas feline information did not consistently improve performance beyond models using wastewater and environmental predictors. This difference may reflect variation in visit volume, geographic coverage, species-specific susceptibility to the pathogens of interest, or the relationship between species-specific syndromic activity and the human outcome. In this study, the value of animal health is demonstrated through comparative predictive modeling, not through an assumption of cross-species transmission.

Current efforts are focused on incorporating data from additional veterinary networks and public health sources to develop a more comprehensive understanding of animal health trends. The stronger predictive signals observed for canine data may partly reflect differences in veterinary utilization, as dogs visit veterinary hospitals at substantially higher rates than cats despite cats being nearly as prevalent as dogs as companion animals in the United States. Feline health records were nevertheless retained to capture potential species-specific responses to environmental or infectious stressors and to avoid excluding potentially informative signals from a major companion-animal population.

The current implementation focuses on county-level data to ensure consistency across data streams. Extending the analysis to finer spatial resolutions, such as the ZIP-code level, remains challenging due to the limited availability and accessibility of ZIP–code–level data, particularly for integrated One Health sources, such as wastewater. Ongoing efforts will address these challenges by seeking higher-resolution and better-aligned datasets to capture localized transmission dynamics and enhance the precision of predictive surveillance.

Additionally, our work showed that the inclusion of multiple random effects (e.g., month, age group, county) enhances model robustness by capturing temporal and demographic variability. To mitigate the risk of overfitting, we carefully validated models using out-of-sample data across multiple counties and time periods, and we report performance metrics such as MAE, RMSE, and correlation to demonstrate consistent predictive accuracy. Future work will explore additional regularization techniques and broader datasets to ensure generalizability across diverse populations and temporal windows.

A key consideration highlighted by our study is the trade-off between interpretability and predictive power across different modeling paradigms. Although Transformer-based architectures can be computationally demanding, the mixed-effects modeling tool developed in this work is lightweight, web-based, and publicly accessible, allowing easy use across regions and institutions without requiring specialized infrastructure or expertise. Mixed-effects models, while requiring more domain knowledge and manual specification, offer a transparent, white-box approach that enables direct examination of model assumptions and variable contributions. This interpretability makes them well-suited for stakeholder communication and policy translation. In contrast, deep neural architectures, such as Transformers, can effectively capture complex, nonlinear patterns and integrate additional spatio-temporal information, enhancing predictive performance—particularly over longer time frames. However, their black-box nature limits interpretability, underscoring the need to pair them with transparent methods to achieve a balance between performance and trustworthiness.

Future extensions of this framework could focus on deeper integration of advanced deep learning approaches with explainable AI (xAI) techniques. Incorporating methods such as LIME or SHAP into the dashboard could help unpack predictions from Transformers and other black-box models, enhancing their usability for decision-makers [52].

Additionally, positioning deep learning approaches as benchmark models within the tool could provide practitioners with reference points for evaluating the adequacy of more interpretable approaches. These directions aim to further bridge the gap between predictive performance and actionable insight, advancing the utility of One Health visual analytics for proactive and transparent syndromic surveillance.

Based on our analyses and cross-case synthesis, we summarized several key lessons that emerge for improving predictive surveillance of infectious disease diffusion in Table 9. These recommendations collectively provide a roadmap for strengthening infectious disease surveillance and outbreak preparedness through integrated, multi-source, and interpretable predictive modeling.

**Table 9:** Lessons Learned and Recommendations for Infectious Disease Surveillance.

Lesson Learned	Recommendation/Best Practice
Multi-source Integration	Combine clinical, environmental, and wastewater data to improve predictive accuracy and early detection; maintain integrated data streams to capture diverse transmission signals.

Incorporating Heterogeneity	Include hierarchical random effects (month, age, county, sex) to capture temporal and demographic variability; critical for reliable forecasting in heterogeneous populations.

Data Quality and Resolution	Address inconsistent reporting and low event counts by investing in higher-resolution and better-aligned datasets; improves local outbreak detection and model generalizability.

Preprocessing and Transformations	Apply STL decomposition and log/SQRT transformations to reduce noise, enhance signal detection, and improve interpretability; regularly evaluate preprocessing steps.

Balancing Performance and Interpretability	Use mixed-effects models for transparency and stakeholder accessibility; use Transformers with spatio-temporal embeddings for longer-horizon forecasts; combine with explainable AI techniques (e.g., SHAP, LIME) to maximize predictive power while maintaining trustworthiness.

Visualization for Decision Support	Provide interactive analytics provenance and visual summaries to allow stakeholders to explore model assumptions, choices, and uncertainties, supporting timely and informed public health interventions.

Policy Implications	Use predictive frameworks to guide proactive resource allocation, outbreak mitigation, and public health messaging; adopt a One Health perspective to address cross-species and environmental drivers of disease.

**Fig. A.5:**
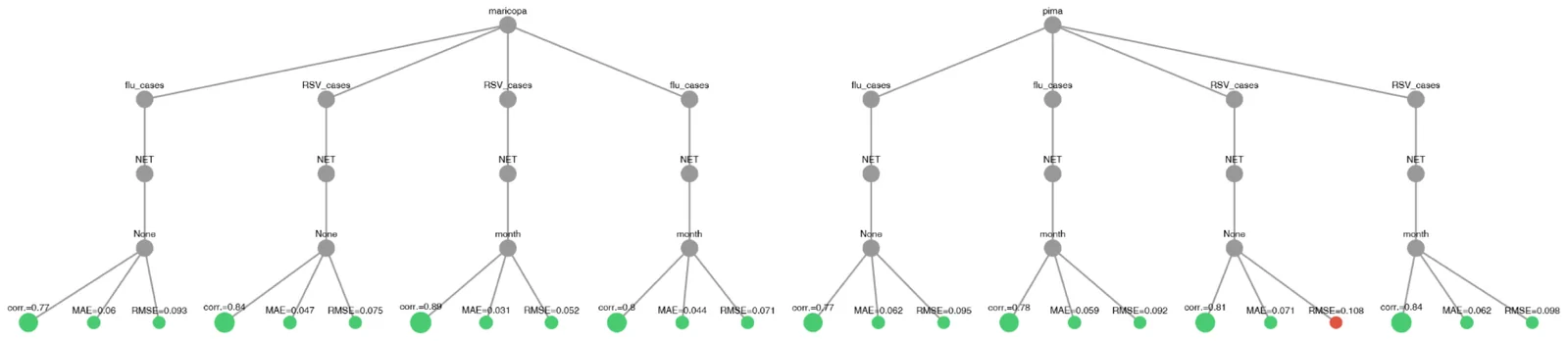
Visual summary of model analytics provenance for comparing two counties, the left tree is for Maricopa County, while the right tree is for Pima County, in the State of Arizona. This figure complements Table 3 by enabling users to visually trace and compare modeling decisions over time.

**Fig. A.6:**
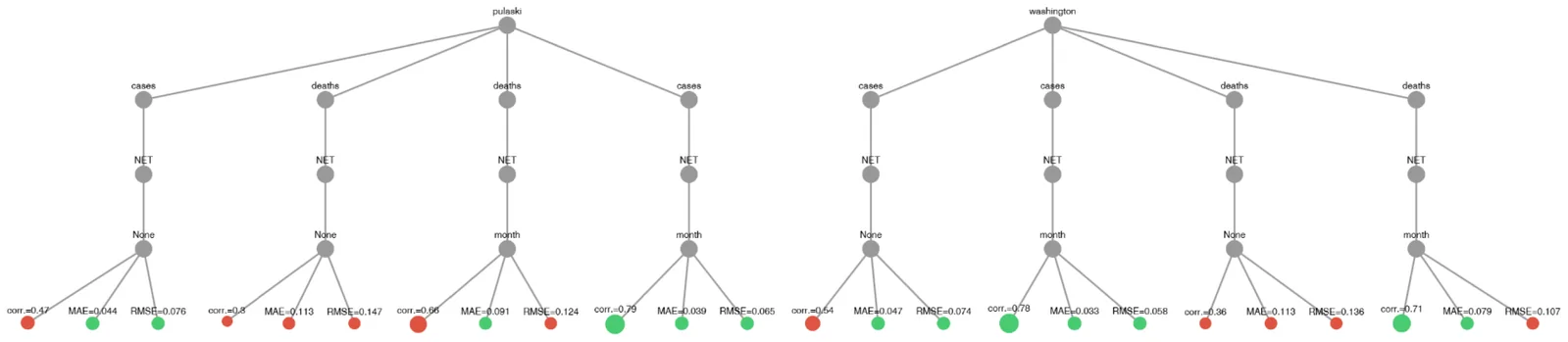
Visual summary of model analytics provenance for comparing two counties, the left tree is for Pulaski County, while the right tree is for Washington County, in the State of Arkansas. This figure complements Table 4 by enabling users to visually trace and compare modeling decisions over time.

## 6 Conclusion

In this study, we present a generalizable One Health visual analytics framework for syndromic surveillance that integrates human clinical data, wastewater monitoring, animal health, and environmental indicators. This framework employs a One Health approach, incorporating relevant variables related to the outcomes we aim to analyze. By combining mixed-effects models, Transformer-based architectures with spatiotemporal embeddings, and interactive dashboards, the framework strikes a balance between predictive accuracy, transparency, and stakeholder interpretability.

• **Theoretical Implications** Our work advances the One Health and syndromic surveillance literature by demonstrating the benefits of multi-source integration for predictive modeling of infectious disease diffusion. Specifically, we demonstrate that hierarchical mixed-effects models, which capture temporal, demographic, and spatial heterogeneity, when combined with deep learning approaches, enhance forecast accuracy while maintaining interpretability. This addresses a critical gap in prior research, which has often relied on single-source data or treated statistical and deep learning methods as separate entities. Moreover, incorporating interactive visual analytics provides a methodological contribution by linking predictive modeling directly with stakeholder-focused interpretability, enabling a bridge between complex models and actionable insights.
• **Managerial and Policy Implications** The proposed framework has direct applications for public health decision-making. Integrating clinical, wastewater, and environmental data enables earlier detection of outbreaks and supports proactive resource allocation. Mixed-effects models offer transparent and explainable results, making them suitable for communication with policymakers, while Transformer-based models provide robust medium- and long-term forecasts to inform planning. By integrating human, environmental, wastewater, and companion animal health surveillance signals, the framework provides a broader evidence base for interpreting infectious-disease patterns and evaluating predictive signals across domains. The observed cross-domain relationships should be interpreted as predictive or associative rather than causal, particularly where evidence is derived from lagged correlations or observational model comparisons. These capabilities facilitate timely interventions, improved outbreak mitigation, and more resilient public health strategies.
• **Ideas for Future Research** Future research directions include extending the framework to finer spatial resolutions, such as ZIP-code or neighborhood-level analysis, to enhance local outbreak detection, while addressing challenges of data availability and alignment. Integrating explainable AI methods (e.g., SHAP or LIME) with deep learning models will improve transparency without compromising predictive performance. Finally, evaluating the framework across multiple regions, diseases, and population demographics will test its generalizability, robustness, and potential for widespread adoption in proactive infectious disease surveillance.

Overall, this work establishes a robust and extensible framework for One Health surveillance, applicable to a wide range of infectious diseases and environmentally linked pathogens, providing a foundation for more resilient and responsive public health decision-making.

## CRediT authorship contribution statement

**Aseel Basheer:** Writing – review & editing, Writing – original draft, Visualization, Software, Resources, Methodology, Formal analysis, Data curation, Conceptualization. **Said Jalal Saidi:** Writing – review & editing, Writing – original draft, Software, Resources, Methodology, Data curation, Conceptualization. **Parisa Masnadi Khiabani:** Writing – review & editing, Writing – original draft, Resources. **Wolfgang Jentner:** Writing – review & editing, Writing – original draft, Visualization, Validation, Resources, Project administration, Investigation, Data curation, Conceptualization. **Aaron Wendelboe:** Writing – review & editing, Writing – original draft, Validation, Resources, Investigation, Data curation. **Jason Vogel:** Writing – review & editing, Writing – original draft, Validation, Resources, Investigation, Data curation. **Katrin Kuhn:** Writing – review & editing, Writing – original draft, Validation, Resources, Investigation, Data curation. **Michael C. Wimberly:** Writing – review & editing, Writing – original draft, Validation, Resources, Investigation, Data curation. **Jo Ann Morrison:** Writing – review & editing, Validation, Resources, Investigation, Data curation. **Kseniya Topdjian:** Writing – review & editing, Validation, Resources, Investigation, Data curation. **Dean Hougen:** Writing – review & editing, Writing – original draft, Validation, Resources, Investigation. **David Ebert:** Writing – review & editing, Writing – original draft, Visualization, Validation, Supervision, Resources, Investigation, Funding acquisition, Data curation, Conceptualization.

## Declaration of competing interest

The authors declare that they have no known competing financial interests or personal relationships that could have appeared to influence the work reported in this paper.

## Data Availability

The code used for data processing, feature engineering, and mixed-effects modeling is publicly available at https://github.com/OU-DISC/mixed-model-creator. The datasets analyzed in this study are available from the corresponding author upon reasonable request, subject to data use agreements and consideration of privacy.

## Appendix Provenance trees

See Fig. A.5, Fig. A.6.

## Footnotes

1 https://mema.disc.ourcloud.ou.edu/.
